# Immature-like molecular expression patterns in the hippocampus of a mouse model of dementia with Lewy body-linked mutant β-synuclein

**DOI:** 10.1186/s13041-018-0378-3

**Published:** 2018-07-06

**Authors:** Hideo Hagihara, Masayo Fujita, Juzoh Umemori, Makoto Hashimoto, Tsuyoshi Miyakawa

**Affiliations:** 1 Division of Systems Medical Science, Institute for Comprehensive Medical Science, Fujita Health University, 1-98 Dengakugakubo Kutsukake-cho, Toyoake, Aichi 470-1192 Japan; 2 Addictive Substance Project, Tokyo Metropolitan Institute of Medical Science, Setagaya-ku, Tokyo, 156-8506 Japan; 3 Laboratory of Parkinson’s Disease, Tokyo Metropolitan Institute of Medical Science, Setagaya-ku, Tokyo, 156-8506 Japan

**Keywords:** β-Synuclein, Endophenotype, Hippocampus, Immature dentate gyrus, Neurodegenerative disorders

## Abstract

**Aim:**

Maturation abnormalities of the brain cells have been suggested in several neuropsychiatric disorders, including schizophrenia, bipolar disorder, autism spectrum disorders, and epilepsy. In this study, we examined the expression patterns of neuronal maturation markers in the brain of a mouse model of dementia with Lewy body-linked mutant β-synuclein (βS), especially in the hippocampus, to explore whether such brain abnormalities occur in neurodegenerative disorders as well.

**Methods:**

Quantitative PCR (qPCR) and immunohistochemical analyses were performed using the hippocampus of 14-month-old P123H βS transgenic (Tg) mice to evaluate the expression of molecular markers for maturation of dentate granule cells.

**Results:**

Based on qPCR results, expression of *Tdo2* and *Dsp* (markers of mature granule cells) was decreased and that of *Drd1a* (a marker of immature granule cells) was increased in the hippocampus of P123H βS Tg mice compared to that in wild-type controls. Immunohistochemical analysis revealed decreased expression of mature granule cell markers Calb1 and Gria1, along with increased expression of the microglial marker Iba1, in the hippocampal dentate gyrus region of P123H βS Tg mice. P123H βS Tg mice exhibited immature-like neuronal molecular expression patterns and microgliosis in the hippocampus. Pseudo-immaturity of dentate granule cells, associated with neuroinflammation, may be a shared endophenotype in the brains of at least a subgroup of patients with neuropsychiatric disorders and neurodegenerative diseases.

**Electronic supplementary material:**

The online version of this article (10.1186/s13041-018-0378-3) contains supplementary material, which is available to authorized users.

## Main text

“Immature dentate gyrus (iDG)” phenotype is commonly found in several mouse models of neuropsychiatric disorders [1], including schizophrenia/intellectual disability [2], bipolar disorder [3], and epilepsy [4, 5]. In this phenotype, almost all granule cells in the adult hippocampal DG are arrested in a pseudo-immature state, in terms of molecular and electrophysiological characteristics. More specifically, molecular features of iDG include a decrease in expression of mature granule cell markers (e.g., tryptophan 2,3-dioxygenase [*Tdo2*], Desmoplakin [*Dsp*], and Calbindin 1 [*Calb1*] [6]) along with an increase in the expression of immature granule cell marker (dopamine receptor D1 [*Drd1a*]) [1]. iDG-like phenotype was also observed in patients with schizophrenia and bipolar disorder [7]. As for neurodegenerative disorders, recent studies revealed that Alzheimer’s disease mouse model showed a drastic decrease in the expression of Calb1 in adult DG, suggestive of iDG-like phenotype in the mouse model [8, 9]. In the present study, we investigated whether a mouse model of dementia with Lewy body-linked mutant β-synuclein (P123H βS) exhibits iDG-related molecular features. The P123H βS transgenic (Tg) mouse showed many behavioral abnormalities, including hyperlocomotor activity, impairment of nest building, and impaired spatial memory, in the middle stage (6–10 months of age), before the onset of motor dysfunction that became apparent in the later stage (12–18 months) [10, 11]. In the brain of these mice, neuritic pathologies such as βS accumulation and axonal swellings, and astrogliosis were observed in various regions, including hippocampus, during the middle to late stage [10]. However, maturation abnormalities in the brain have not been examined in these mice.

**Fig. 1:**
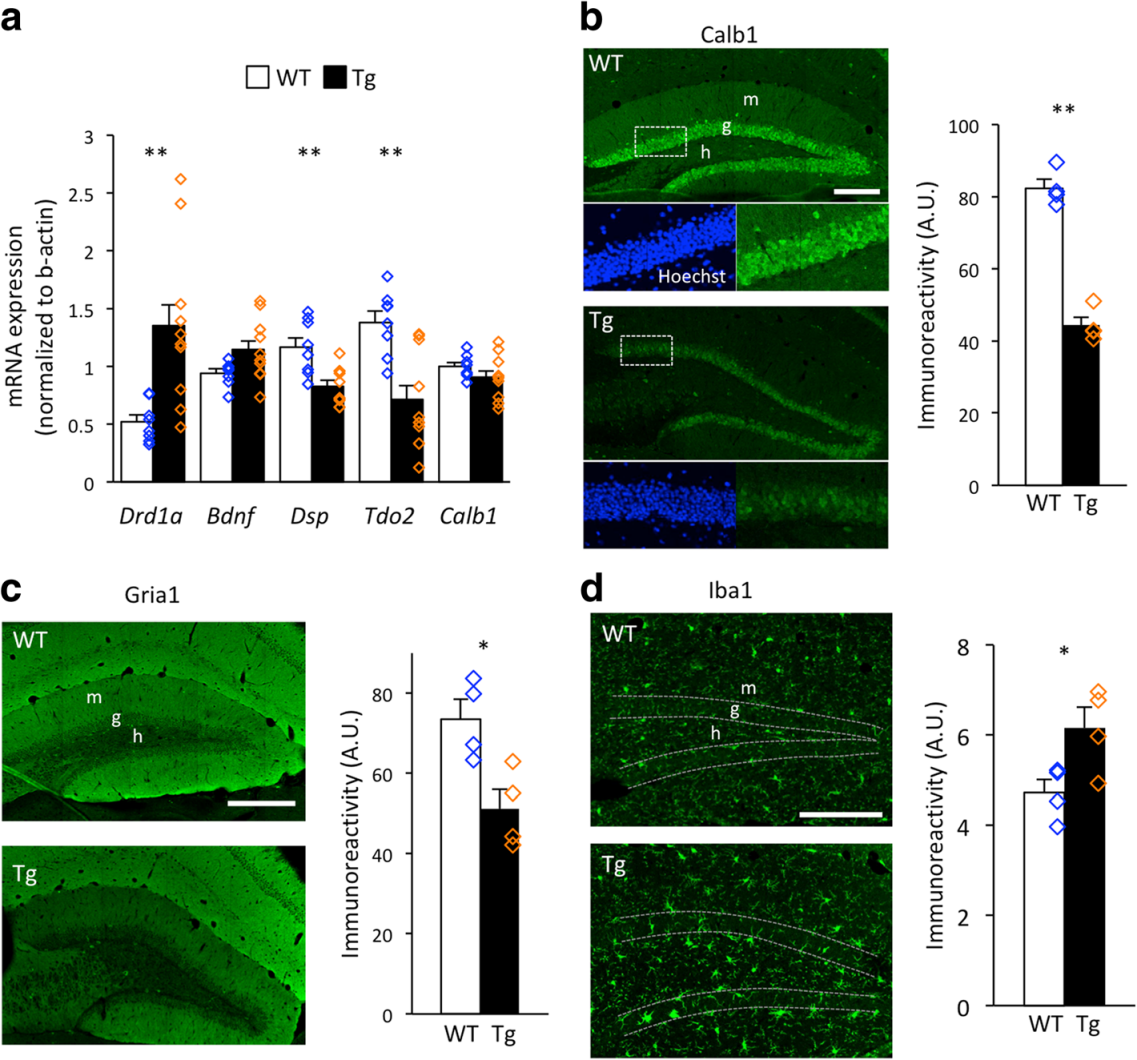
iDG-like molecular phenotypes in the hippocampus of P123H βS Tg mice. **a** Results of quantitative PCR. Bar graphs represent relative mRNA expression levels normalized to *β-actin* mRNA. Data obtained from two independent experiments were combined and shown as the mean ± SEM. (*n* = 8 for wild-type mice and *n* = 10 for P123H βS Tg mice). *P < 0.05, **P < 0.01 versus wild-type mice*.* **b**–**d** Representative images (*left panels*) and the semi-quantitative results (*right panels*) of immunofluorescence imaging of Calb1 (**b**), Gria1 (**c**), and Iba1 (**d**) in the DG of wild-type and P123H βS Tg mice. Higher magnification of the boxed area is shown below the corresponding panel (**b**). Data are shown as the mean ± SEM (*n* = 4 for each genotype). *P < 0.05, **P < 0.01 versus wild-type mice*.* Scale bars, 200 μm (**b**, **d**) and 500 μm (**c**). g, granule cell layer; h, hilus; m, molecular layer

Whole hippocampus of the mouse (14 months of age) was dissected out and quantitative PCR (qPCR) analysis was conducted to examine mRNA expression levels of *Drd1a*, *Bdnf*, *Tdo2*, *Dsp*, and *Calb1*. The detailed method for qPCR is described in the Additional file 1. Expression of *Drd1a* was significantly increased and that of *Bdnf* showed an increasing trend in the hippocampus of P123H βS Tg mice compared to that in wild-type mice (Fig. 1a, Additional file 1: Table S1). Expression of *Tdo2* and *Dsp* was significantly decreased in the P123H βS Tg mice while that of *Calb1* was comparable between P123H βS Tg and wild-type mice. To assess Calb1 expression, focusing on the DG region, we conducted immunohistochemical analyses (see Additional file for the detailed method). The results showed a significant decrease in Calb1 expression in the DG granule cell layer of P123H βS Tg mice (Fig. 1b, Additional file 1: Figure S1). Notably, a patch-like reduction of Calb1 immunoreactivity was observed in the DG granule cell layer of P123H βS Tg mice, which may not have been due to apparent cell loss, since nuclear staining was observed uniformly throughout the DG granule cell layer. We also found a decrease in immunoreactivity for Gria1, whose expression increased with maturation of granule cells [12], but decreased in the DG of typical mouse models with iDG [12], and in that of P123H βS Tg mice (Fig. 1c). In addition, immunoreactivity for ionized calcium binding adaptor molecule 1 (Iba1) was increased in the DG of P123H βS Tg mice (Fig. 1d), suggesting that microglia are activated in the DG of these mice.

We found a significant decrease in Calb1 expression in the DG granule cell layer by immunohistochemical analysis. However, the extent of this reduction was low relative to that found in other mouse models with iDG, such as *Camk2a*^+/−^ mice, *Shn2* KO mice, and mutant *Snap25* knock-in mice, whose Calb1 expression in the DG granule cell layer was almost completely depleted [2–4]. Therefore, the present qPCR analysis of whole hippocampus samples might have failed to detect a decrease in *Calb1* expression in P123H βS Tg mice, due to the presence of cells (other than granule cells) that express *Calb1*, such as pyramidal cells in the Ammon’s horn region and particular types of interneurons that exist throughout the brain. The discrepancy between mRNA and protein levels of Calb1 may also be accounted for by some post-transcriptional mechanisms and/or differences in their half lives [13].

Patch-like reduction of Calb1 expression in the DG granule cell layer was found in P123H βS Tg mice; a similar phenotype was observed in a mouse model of Alzheimer’s disease (line J20) [8, 9]. In those papers, it was suggested that Calb1 downregulation was induced by seizure activity in patients and mouse models [8, 9]. Patch-like reduction of Calb1 in the DG has been observed in epilepsy models [5]. Considering that epileptic seizures have been observed in P123H βS Tg mice (unpublished observation), seizure activity might have caused Calb1 downregulation in these Tg mice. Interestingly, patch-like reduction of Calb1 in the DG have also been found in adult mice treated with antidepressant fluoxetine [14] and electroconvulsive stimulation [15]. Assuming that typical models showing robust depletion of Calb1, such as *Camk2a*^+/−^ and *Shn2* KO mice [2, 3], display iDG phenotypes developmentally generated, there is the possibility that these weaker phenotypes are features of iDG that are induced by dematuration in adults.

In conclusion, P123H βS Tg mice exhibited iDG-like signatures and microgliosis in the DG. It would be of interest to determine the time of appearance of the iDG phenotype in these mice in relation to that of behavioral abnormalities.

## Additional file

Additional file 1: Materials and Methods. **Table S1.** Raw data of qPCR analysis (average *C*q value). **Figure S1.** Immunohistochemical images used for quantitative analysis in this study. (DOCX 25253 kb)

## Abbreviations

Bdnf: Brain-derived neurotrophic factor
Calb1: Calbindin
Camk2a: Calcium/calmodulin-dependent protein kinase II alpha
DG: Dentate gyrus
Drd1a: Dopamine receptor D1
Dsp: Desmoplakin
Gria1: Glutamate receptor, ionotropic, AMPA 1
Iba1: Ionized calcium binding adaptor molecule 1
iDG: Immature dentate gyrus
PBS: Phosphate-buffered saline
PCR: Polymerase chain reaction
Shn2: Schnurri-2
Snap25: Synaptosomal-associated protein, 25 kDa
Tdo2: Tryptophan 2,3-dioxygenase
βS: β-synuclein
