# Supplementary material for: Immature-like molecular expression patterns in the hippocampus of a mouse model of dementia with Lewy body-linked mutant β-synuclein
Source: Mol Brain. 2018 Jul 6;11:38. doi: 10.1186/s13041-018-0378-3 (PMC6034225; doi:10.1186/s13041-018-0378-3)
Supplement: Supplementary file 1 — Materials and Methods. Table S1. Raw data of qPCR analysis (average Cq value). Figure S1. Immunohistochemical images used for quantitative analysis in this study. (DOCX 25253 kb) [file 13041_2018_378_MOESM1_ESM.docx]

Additional file 1

**Immature-like molecular expression patterns in the hippocampus of a mouse model of dementia with Lewy body-linked mutant β-synuclein**

Hideo Hagihara, Masayo Fujita, Juzoh Umemori, Makoto Hashimoto, Tsuyoshi Miyakawa*

*Corresponding author. E-mail: miyakawa@fujita-hu.ac.jp

**This file includes:**

Materials and Methods

Table S1

Figure S1

**Materials and Methods**

**Quantitative real-time PCR**

Whole hippocampus was dissected out, immediately frozen in liquid nitrogen, and stored at –80 °C until use. Total RNA was isolated from the hippocampi of wild-type and P123H βS Tg mice. First-strand cDNA was synthesized from 1 μg of DNase I-treated total RNA using the Superscript® VILO™ cDNA synthesis kit (Life Technologies, Grand Island, NY). The expression of related genes was quantified using SYBR GreenER qPCR SuperMix for ABI PRISM (Life Technologies), following the manufacturer’s instructions. Quantitative PCR was performed using ABI PRISM 7700 (Life Technologies) with the following conditions: 2 min at 50 °C and 10 min at 95 °C, followed by 40 cycles of 15 s at 94 °C and 1 min at 60 °C. β-actin was amplified from all the samples to normalize expression levels. The following primers were used:

*β-actin*: 5ʹ- agtgtgacgttgacatccgta and 5 ʹ - gccagagcagtaatctccttct;

*Bdnf*: 5 ʹ - tcatacttcggttgcatgaagg and 5 ʹ - agacctctcgaacctgccc;

*Calb1*: 5 ʹ - gaatcccacctgcagtcatctc and 5 ʹ - tggatcaagttctgcagctcc;

*Drd1a*: 5 ʹ - gccgctgtcatcaggtttc and 5 ʹ - ggccaaaagccagcaatct;

*Dsp*: 5 ʹ - gctgaagaacactctagccca and 5 ʹ - actgctgtttcctctgagaca;

*Tdo2*: 5 ʹ - atgagtgggtgcccgtttg and 5 ʹ - ggctctgtttacaccagtttgag.

**Immunohistochemistry**

Fourteen-month-old mice (*n* = 4 for each genotype) were anesthetized and transcardially perfused with 4 % paraformaldehyde in phosphate-buffered saline (PBS). The brains were dissected, immersed overnight in the same fixative, and transferred to 30 % sucrose in PBS for at least three days for cryoprotection. Brains were mounted in Tissue-Tek (Miles, Elkhart, IN), frozen, and cut into 8-μm-thick coronal sections using a microtome (CM1850; Leica Microsystems, Wetzlar, Germany). The sections were pre-incubated for 30 min at room temperature in 5 % skim milk in PBS containing 0.05 % Tween-20 (PBST), and then incubated overnight at 4 °C in PBS containing the primary antibodies. We used the following primary antibodies: mouse monoclonal anti-Calb1 antibody (300; Swant, Bellinzona, Switzerland), rabbit polyclonal anti-Gria1 antibody (AB1504; Millipore, Temecula, CA), and rabbit polyclonal anti-Iba1 antibody (019-19741; Wako Pure Chemical Industries, Osaka, Japan). Immunoreactivity to the antigen was visualized using Alexa488-conjugated secondary antibody (Molecular Probes, Eugene, OR). Nuclear staining was performed with Hoechst 33258 (Polysciences, Warrington, PA). We used a confocal microscope (LSM 510 META; Zeiss, Göttingen, Germany) to obtain images of the stained sections. Quantification of immunofluorescence intensities was performed using ZEN software (Zeiss). One section, obtained from the dorsal DG, was examined per animal. Dentate gyrus regions were delineated manually on the Hoechst-stained images (areas with visible cracks were avoided). In Iba1-immunoreactivity quantification, fluorescence intensity of the area near the cellular boundary was used to subtract background noise.

**Table S1.** Raw data of qPCR analysis (average *C*q value).

|  | Cohort | Genotype | *β-actin* | *Bdnf* | *Calb1* | *Drd1a* | *Dsp* | *Tdo2* |
| --- | --- | --- | --- | --- | --- | --- | --- | --- |
| 1 | 1 | WT | 426.2 | 412.1 | 495.6 | 230.3 | 588.3 | 650.1 |
| 2 | 1 | WT | 531.4 | 833.5 | 336.9 | 1392.1 | 508.0 | 65.0 |
| 3 | 1 | WT | 577.0 | 526.8 | 630.7 | 185.8 | 818.5 | 906.9 |
| 4 | 1 | WT | 592.7 | 435.1 | 554.9 | 260.9 | 872.0 | 812.4 |
| 5 | 1 | WT | 563.7 | 552.2 | 584.8 | 197.4 | 667.8 | 1002.6 |
| 6 | 1 | Tg | 458.5 | 603.8 | 334.1 | 546.9 | 439.3 | 378.3 |
| 7 | 1 | Tg | 438.5 | 672.0 | 532.9 | 675.3 | 281.7 | 144.1 |
| 8 | 1 | Tg | 468.4 | 343.4 | 537.7 | 374.0 | 331.5 | 593.1 |
| 9 | 1 | Tg | 551.9 | 609.3 | 508.0 | 767.2 | 395.1 | 281.5 |
| 10 | 1 | Tg | 592.3 | 622.0 | 514.8 | 692.1 | 511.3 | 304.0 |
| 11 | 2 | WT | 538.5 | 575.4 | 463.7 | 213.2 | 454.7 | 817.2 |
| 12 | 2 | WT | 582.8 | 583.9 | 550.7 | 443.3 | 638.8 | 736.6 |
| 13 | 2 | WT | 618.5 | 536.9 | 570.7 | 351.5 | 601.8 | 661.0 |
| 14 | 2 | WT | 593.3 | 592.0 | 599.6 | 452.4 | 559.0 | 555.4 |
| 15 | 2 | Tg | 631.2 | 645.2 | 576.3 | 396.1 | 594.1 | 779.0 |
| 16 | 2 | Tg | 679.7 | 850.0 | 462.1 | 1637.6 | 436.4 | 323.0 |
| 17 | 2 | Tg | 564.9 | 529.4 | 509.1 | 265.1 | 629.9 | 722.5 |
| 18 | 2 | Tg | 544.6 | 507.8 | 564.7 | 695.7 | 394.6 | 302.9 |

Tg, P123H βS Tg mice; WT, wild-type mice.

**Figure S1. Immunohistochemical images used for quantitative analysis in this study.** Calb1 (**a**), Gria1 (**b**), and Iba1 (**c**) staining in the hippocampus of wild-type (*left panels*) and P123H βS Tg mice (*right panels*). Scale bars, 300 μm.
